# Liutex-Represented Vortex Spectrum in Turbulence

**DOI:** 10.3390/e25010025

**Published:** 2022-12-23

**Authors:** Bowen Yan, Yiqian Wang, Chaoqun Liu

**Affiliations:** 1 Department of Computer Science and Technology, Tsinghua University, Beijing 100084, China; 2 School of Mathematical Science, Soochow University, Suzhou 215006, China; 3 Department of Mathematics, University of Texas at Arlington, Arlington, TX 76019, USA

**Keywords:** Liutex, vortex, similarity law, turbulence, homogeneous, isotropic turbulence, turbulent channel

## Abstract

The Liutex vector is new quantity introduced to represent the rigid-body rotation part of fluid motion and thus to define and identify vortices in various flows. In this work, the intermittency and power-law similarity of the Liutex vector in homogeneous, isotropic turbulence and a turbulent channel are explored. First, we found that the Liutex vector is more intermittent than the vorticity vector in the considered turbulent flows, which indicates that an iso-surface of a Liutex magnitude with an appropriate threshold could capture the major rotating motions or vortical motions of the flow. Second, the three-dimensional energy spectrums of velocity, vorticity (enstrophy spectrum) and the Liutex vector in homogeneous isotropic turbulence are shown to exhibit power laws of −5/3, 1/3 and 1/3 in the inertial subrange, respectively, whilst the Liutex energy spectrum particularly satisfies an additional −10/3 power law in the viscous subrange. This viscous similarity of the Liutex vector is the only power law that survived from the wall presence and is argued to originate from the fact that the Liutex vector represents the rigid part of fluid motion and is free from any shear contamination. The existence of such a viscous similarity law indicates a certain coherence of the small scales of turbulence and could possibly help understand and model turbulence.

## 1. Introduction

Turbulence consists of numerous vortices. Therefore, to define—and thus, to identify—vortices is of great importance in understanding turbulence structures and mechanisms. A new vector, named Rortex and later renamed Liutex, has been introduced, whose direction is the local rotation axis and magnitude is twice the angular velocity of the rigid rotation part of fluid motion [1,2]. The definition of the Liutex vector is purely kinematic, i.e., based on the velocity gradient tensor $\nabla \vec{V}$. Given that $\nabla \vec{V}$ has one real eigenvalue, $\lambda_{r}$, with a corresponding real unit eigenvector ${\vec{v}}_{r}$ and two complex-conjugate eigenvalues $\lambda_{cr}\pm \lambda_{ci}$, otherwise $\nabla \vec{V}$ has three real eigenvalues and no rotational motion could be found, the rotational axis has to be in the same direction as the real eigenvector. It is argued that only compression and stretch can be found in the direction of the real eigenvector; thus, any rotational motion has to be in the plane perpendicular to the real eigenvector. Therefore, the direction of the Liutex vector is obtained by $\vec{r}={\vec{v}}_{r}$. Both ${\vec{v}}_{r}$ and $-{\vec{v}}_{r}$ are unit eigenvectors corresponding with the eigenvalue $\lambda_{r}$. To uniquely define the Liutex direction, we require that $\vec{\omega }\cdot \vec{r}>0$, with $\vec{\omega }$ as the vorticity vector. In the next step, we determine the magnitude of the Liutex vector by coordinate rotation. First, we rotate the coordinates so that the new *z*-axis is in the direction of $\vec{r}$. We then rotate the coordinates around the new *z*-axis, i.e., $\vec{r}$, and the rotational strength is defined as twice the minimal absolute value of the off-diagonal component of the 2×2 upper left submatrix of the velocity gradient tensor. The resulting coordinate is referred to as the principal coordinate in this paper and the quantities evaluated in this coordinate are denoted by a superscript asterisk. The velocity gradient tensor in the principal coordinate had the form:

(1) $$\nabla \vec{V^{*}}=\left[\begin{array}{ccc} \lambda_{cr} & -\phi & 0\\ \phi +s & \lambda_{cr} & 0\\ \xi & \eta & \lambda_{r} \end{array}\right],$$

thus, $\nabla \vec{V^{*}}$ could be decomposed as:

(2) $$\nabla \vec{V^{*}}=\left[\begin{array}{ccc} 0 & -\phi & 0\\ \phi & 0 & 0\\ 0 & 0 & 0 \end{array}\right]+\left[\begin{array}{ccc} \lambda_{cr} & 0 & 0\\ 0 & \lambda_{cr} & 0\\ 0 & 0 & \lambda_{r} \end{array}\right]+\left[\begin{array}{ccc} 0 & 0 & 0\\ s & 0 & 0\\ \xi & \eta & 0 \end{array}\right]\equiv \vec{R^{*}}+\vec{C^{*}}+\vec{S^{*}},$$

where $\vec{R^{*}}$, $\vec{C^{*}}$ and $\vec{S^{*}}$ represent the local rotational part, the compression/stretching part and the pure shear part of fluid motion, respectively. Here, ϕ represents the angular velocity of the rigid rotation part of fluid motion whilst s, ξ and η are the residual shear along the three spatial dimensions. Different from Cauchy–Stokes decomposition, which splits the velocity gradient tensor into a symmetric part and an antisymmetric part, this decomposition extracts the pure rotation part, which would not be countervailed by the residual compression/stretching and shear. The magnitude of the Liutex vector, i.e., the rotational strength, is thus defined as twice the angular velocity of the rotational part:

(3) R=2ϕ,

Therefore, the Liutex vector in the principal and original coordinates could be written as $\vec{R^{*}}={\left[\begin{array}{ccc} 0 & 0 & R \end{array}\right]}^{T}$ and $\vec{R}=R\vec{r}$, respectively. It is easy to verify in the principal coordinate that the vorticity vector could be decomposed as the sum of the Liutex vector and the pure shear vector, i.e.,

(4) $$\vec{\omega^{*}}=\vec{R^{*}}+\vec{S^{*}},$$

with:

(5) $$\vec{\omega^{*}}=\left[\begin{array}{c} \eta \\ -\xi \\ 2\phi +s \end{array}\right]\;\vec{R^{*}}=\left[\begin{array}{c} 0\\ 0\\ 2\phi \end{array}\right]\;\vec{S^{*}}=\left[\begin{array}{c} \eta \\ -\xi \\ s \end{array}\right],$$

Note that the symbols $\vec{R}$ and $\vec{S}$ have been used to represent the rotation part and shear part, respectively, both in the vector form and the tensor form to highlight their correspondence between the two forms. In addition, as the vorticity vector and the Liutex vector are Galilean-invariant [3], i.e., invariant under coordinate transformation and rotation, Equation (4) could be projected into the original coordinates as $\vec{\omega }=\vec{R}+\vec{S}$, which is called the RS decomposition of vorticity [4].

Although the coordinate rotation approach to calculate the Liutex vector is more intuitive, especially in prompting the systematic decomposition of fluid motion in the tensor form (Equation (2)) and vector form (Equation (4)), an explicit formula was derived that is both physically revealing and is proved to be more efficient in terms of computation efficiency [5]. It was found, by studying the flow pattern induced by the given velocity gradient tensors, that the vorticity in the direction of the rotational axis could be viewed as twice the spatial average angular velocity; on the other hand, the imaginary part of the complex-conjugate eigenvalue could be viewed as the pseudo-time average angular velocity. Combining these two relations, the minimum angular velocity, i.e., the Liutex magnitude R, could be obtained; thus, the Liutex vector could be written as:

(6) $$\vec{R}=R\vec{r}=\left(\vec{\omega }\cdot \vec{r}-\sqrt{{\left(\vec{\omega }\cdot \vec{r}\right)}^{2}-4\lambda_{ci}^{2}}\right)\vec{r},$$

which is an explicit formula in the original coordinate to calculate the Liutex vector in terms of the vorticity vector $\vec{\omega }$, the imaginary part of the complex-conjugate eigenvalue $\lambda_{ci}$ and the real eigenvector of the velocity gradient tensor $\vec{r}$. With Equation (6), we could decompose vorticity $\vec{\omega }$ into $\vec{R}$ and $\vec{S}$ in the original coordinate without explicitly performing a reference frame rotation.

Compared with previous vortex identification methods, the Liutex system has two distinctive features. First, the directional information is included by introducing a vector quantity $\vec{R}$**,** with its direction as the local rotation axis. Intuitively, any rotational motion should have an axis to rotate about, which is generally ignored. Second, the magnitude of the Liutex vector represents the local angular speed of the rigid rotation part of fluid motion; thus, it is free from shear contamination. Six critical aspects of vortex identification, including the absolute strength, relative strength, local rotational axis, global rotation axis, vortex core size and vortex boundary, can be extracted from the flow field based on the Liutex system [4,6]. Multiple vortex identification strategies have been introduced based on the Liutex vector, for example, the Liutex magnitude iso-surface, objective Liutex [7], the Liutex-Ω method [8,9] and the Liutex core line method [10,11]; these methods have been proven to be effective in capturing vortices in various flows [12,13].

Another notable feature of the Liutex vector is that the streamwise spectrum of the Liutex magnitude follows the −5/3 power law in the buffer layer of boundary-layer turbulence [14], which reveals that turbulence has structures down to a viscous scale. To better understand the power-law spectrum of Liutex in turbulence, we used data of homogeneous isotropic turbulence (HIT) with $Re_{\lambda }=315$ and a turbulent channel flow with $Re_{\tau }=950$. The rest of the paper is organized as follows. Section 2 introduces the numerical aspects of homogeneous isotropic turbulence. And the data from a turbulent channel of $Re_{\tau }=950$ are also used to illustrate the universality of the Liutex similarity. Section 3 focuses on the distribution of the Liutex vector in homogeneous isotropic turbulence, with an emphasis on the power-law spectrum of Liutex. Finally, conclusions are given in Section 4.

## 2. Case Set-Up, Data and Numerical Strategy

DNS data of homogeneous isotropic turbulence with $Re_{\lambda }=315$ made public by Cardesa et al. [15] were used. The numerical data were obtained by solving the incompressible Navier–Stokes equations with a pseudo-spectral treatment. The computational domain of the dataset was a triply periodic cube with a side length of 2π resolved by 1024 points along each direction. Deterministic forcing at the two lowest wave numbers was adopted to make the flow statistically stationary. The spatial resolution was set to $k_{max}\eta =2$, where $k_{max}$ and η denote the maximum resolved wavenumber and Kolmogorov scale, respectively. Thus, the computational domain of the datasets covered ${\left(1516\eta \right)}^{3}$ in space and lasted 2090 small-scale time units ($\tau =\sqrt{\nu /\varepsilon }$, where ν is the kinematic viscosity and ε is the dissipation rate of the turbulent kinetic energy).

Even though the database was temporally resolved in the sense that, statistically, the characterization of a phenomenon throughout its life is tractable [15], the temporal resolution was not enough to properly approximate the Eulerian derivatives of the Liutex vector and other quantities. To overcome this issue, we selected one of the last snapshots and advanced it in time with the forward Euler method by Δt=0.00078τ, which equaled one-hundredth of the time interval between the saved snapshots in the database. The convergence in approximating the Eulerian derivatives was verified by using an even smaller Δt. The spatial discretization basically followed the algorithms presented by Rogallo [16] and the 3/2 rule was used to avoid an aliasing error. The physical model behind the algorithm was that sustained homogeneous isotropic turbulence was first obtained (the database) and then we allowed the turbulence to freely decay by solving the Navier–Stokes equation without forcing the term, thus obtaining the Eulerian derivatives. In this way, we could study how the Liutex vector evolved in homogenous isotropic turbulence. The numerical data of a turbulent channel with $Re_{\tau }=950$ [17,18] was also used in this study, mostly to reveal the universality of the Liutex similarity over other spectrums such as the −5/3 power spectrum of turbulent kinetic energy [19].

## 3. Liutex Similarity in Turbulence

**Figure 1:**
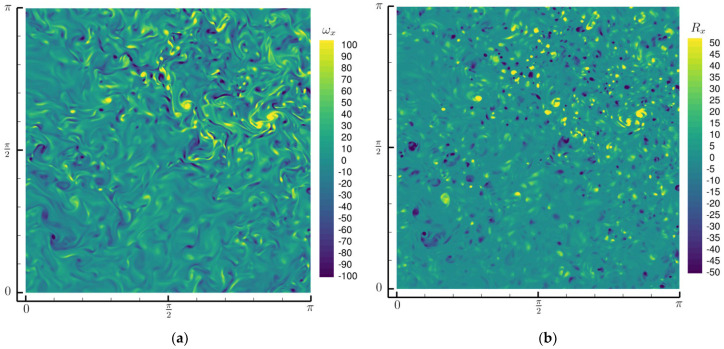
Distributions on a selected plane of x=π/2. (**a**) The x—direction vorticity component; (**b**) the x—direction Liutex component.

**Figure 2:**
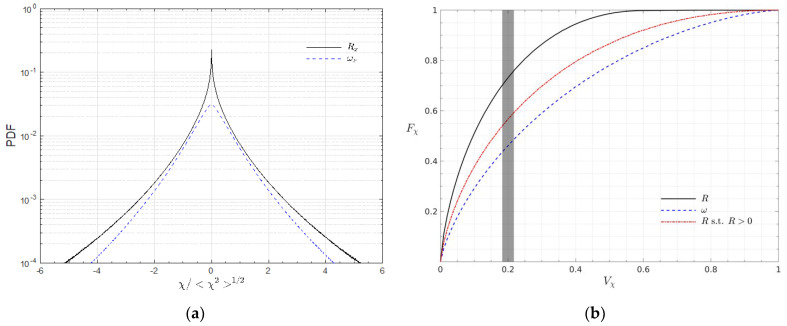
(**a**) Probability density function of $R_{x}$ and $\omega_{x}$; (**b**) cumulative property fraction $F_{\chi }$ against cumulative probability $V_{\chi }$.

Figure 1 shows the distribution of the vorticity component $\omega_{x}$ and the Liutex component $R_{x}$ on the part of the plane x=π/2. Obviously, $R_{x}$ is more concentrated on the islands of high magnitude that are separated by regions of $R_{x}=0$ whereas $\omega_{x}$ is more continuously distributed across the plane. A simple calculation reveals that 40% of the whole computational volume is void of Liutex, i.e., only 60% of the domain has a Liutex magnitude larger than 0. Intuitively, the vortices are generally concentrated phenomena, which seems to agree better with the distribution of Liutex. It should be noted that the magnitude of Liutex was always smaller than the vorticity, based on Equation (6). However, this relation does not extend directly to the component because the directions of the Liutex and vorticity are generally different. For example, about 7% of the whole domain in this snapshot is equipped with a $\left|R_{x}\right|$ larger than $\left|\omega_{x}\right|$. Nonetheless, we could claim that for homogeneous isotropic turbulence, the magnitude of the vorticity component is generally larger than that of the same Liutex component as this was true for the other 93% of the volume. It can be observed from Figure 1b that, generally, an island with a positive $R_{x}$ concentration (counter-clockwise rotating from the perspective of Figure 1b) is surrounded by islands of multiple negative $R_{x}$ concentrations (clockwise rotating) and vice versa. The probability density functions (PDFs) of $R_{x}$ and $\omega_{x}$ are shown in Figure 2a. Note that there was only 60% volume when R>0 was taken into account when obtaining the PDF of $R_{x}$. Even without taking the area of R=0 into account, we could see that the PDF of $R_{x}$ is significantly more concentrated near 0 than that of $\omega_{x}$; i.e., compared with $\omega_{x}$, $R_{x}$ has a greater probability of being near zero and seldom excurses to large values. In total, 33% of the non-zero $R_{x}$ is in the range $\left(-\langle R_{x}^{2}\rangle {}^{\frac{1}{2}},\langle R_{x}^{2}\rangle {}^{\frac{1}{2}}\right)$ (nearly 60% of the whole volume if the zeros were taken into account) whereas only 19% of $\omega_{x}$ is in the range $\left(-\langle \omega_{x}^{2}\rangle {}^{\frac{1}{2}},\omega_{x}^{2}{}^{\frac{1}{2}}\right)$.

**Figure 3:**
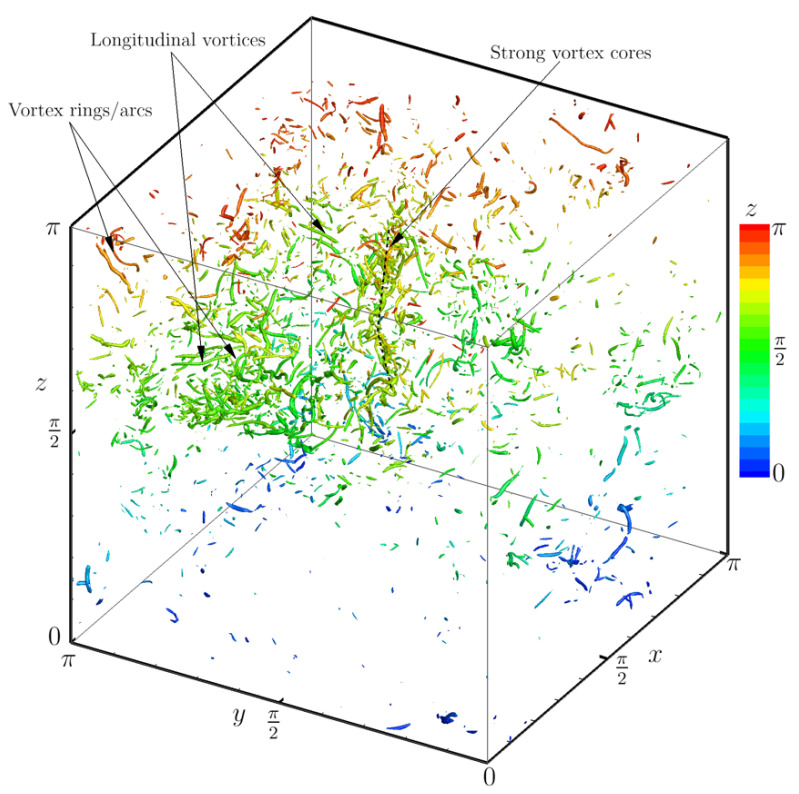
Iso-surfaces of $R=18R^{2}{}^{1/2}$ to represent vortical structures.

Despite the general coincidences of the local magnitude maximum in Figure 1, the distributions of $\omega_{x}$ and $R_{x}$ are rather different. The term “intermittency” is often used to refer to the uneven scattering of turbulent fluctuations both in space and time, and has been argued to be a reflection and indicator of coherent structure motion [20]. Here, we adopt the definition by Jiménez [21]. For a quantity χ with a probability density function (PDF) of p(χ), the volume fraction of the data above a threshold $\chi_{0}$ is defined as:

(7) $$V_{\chi }\left(\chi_{0}\right)=\int_{\chi_{0}}^{\infty }p\left(\chi \right)d\chi ,$$

and the fraction of χ above that threshold is defined as:

(8) $$F_{\chi }\left(\chi_{0}\right)={\langle \chi \rangle }^{-1}\int_{\chi_{0}}^{\infty }\chi p\left(\chi \right)d\chi ,$$

with 〈χ〉 as the average defined by 〈χ〉=∫χp(χ)dχ; thus, $F_{\chi }\left(\chi_{0}\right)$ could be viewed as the fraction of the contribution from χ greater than $\chi_{0}$ to the average 〈χ〉. If a threshold $\chi_{0}$ could be obtained such that $V_{\chi }\left(\chi_{0}\right)$ is relatively small whilst $F_{\chi }\left(\chi_{0}\right)$ is relatively large—i.e., the average 〈χ〉 is relatively determined by a small fraction of concentrated large χ—the variable χ is then considered to be intermittent. Figure 2b provides the cumulative property fraction $F_{\chi }$ against the cumulative probability $V_{\chi }$ of the vorticity magnitude ω and the Liutex magnitude R, with or without accounting for R=0 areas. Obviously, R is more intermittent than ω. As indicated by the grey patch, we can expect the isolation of intense structures, based on R accounting for approximately 75% of the energy with a relatively small volume fraction of 20%. Ignoring the zero values of R leads to a decrease in the cumulative property fraction $F_{\chi }$ to 55% for the volume fraction $V_{\chi }$ of 20%, which is still higher than that of the vorticity magnitude ω at 45%. The reason that the $F_{\chi }$ of R approaches 1 as $V_{\chi }$ approaches 0.6 is again that 40% of the R values are zero. The intermittency of the Liutex magnitude indicates that strong rotational motion is concentrated in a small volume fraction; thus, an iso-surface of the Liutex magnitude with an appropriate threshold could capture the majority of the vortical structures. Iso-surfaces of $R=8\langle R^{2}\rangle {}^{1/2}$ colored by z are shown in Figure 3 to represent the vortices in the flow. The strong vortex cores, vortex rings/arcs and longitudinal vortices, which are typically found in turbulence, are well-captured. Intermittency of the Liutex magnitude or the vortices are observed; a greater number of vortices are found in the upper half (z>π/2) than in the lower half (z>π/2) in the shown domain. Those vortices could be viewed as skeletons of the flow and served as the build-up elements of turbulence.

**Figure 4:**
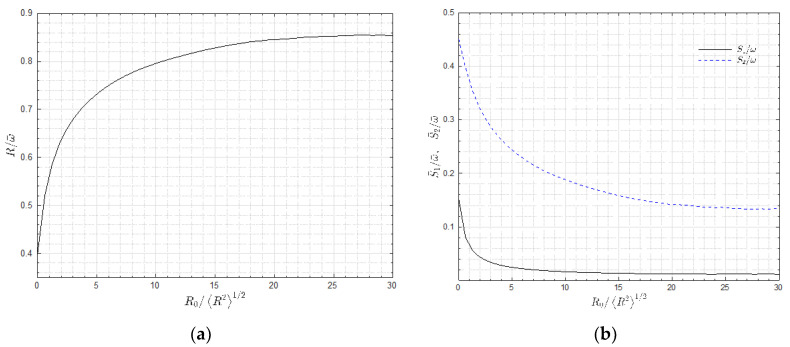
(**a**) The ratio of $\bar{R}$ over $\bar{\omega }$ as selected threshold $R_{0}$; (**b**) non-alignment shear ${\bar{S}}_{1}$ and in-plane residual shear ${\bar{S}}_{2}$.

To study the relationship between the Liutex and vorticity inside the vortices, especially the vortex cores, we define the partial average $\bar{R}$ and $\bar{\omega }$ as functions of a selected threshold, $R_{0}$:

(9) $$\bar{R}\left(R_{0}\right)=mean\left(\left\{R|R\rangle R_{0}\right\}\right),$$

(10) $$\bar{\omega }\left(R_{0}\right)=mean\left(\left\{\omega |R\rangle R_{0}\right\}\right),$$

thus, the ratio $\bar{R}/\bar{\omega }$ defines the rigidity of the partial volume $R>R_{0}$ as $\bar{R}/\bar{\omega }$ equals 1 if the fluid rotates similar to a rigid body and equals 0 if the fluid does not rotate at all. The calculated ratio $\bar{R}/\bar{\omega }$ of the considered homogeneous isotropic turbulence is shown in Figure 4a. At $R_{0}=0$, i.e., considering the volume with R>0, the ratio of the mean rotational strength over the mean vorticity magnitude equals 0.4. As $R_{0}$ increases, the volume to obtain an average became smaller and the ratio quickly increases and saturates at around 0.85 at $R_{0}\;\approx \;20\langle R^{2}\rangle {}^{1/2}$; above this, the area could be considered to be the vortex core region. Note that a similar threshold has been selected in Figure 3 to represent the vortices; thus, we could assume that the iso-surface enclosed volumes are vortex cores. According to Equation (6), the fact that the Liutex magnitude is smaller than the vorticity magnitude arise from two mechanisms: first, the non-alignment of the Liutex vector and the vorticity vector, expressed as $S_{1}=\omega -\vec{\omega }\cdot \vec{r}$; and second, the residual shear in the rotational plane $S_{2}=\sqrt{{\left(\vec{\omega }\cdot \vec{r}\right)}^{2}-4\lambda_{ci}^{2}}$. With these definitions, we could obtain $R=\omega -S_{1}-S_{2}$. Figure 4b shows the similar defined partial average ${\bar{S}}_{1}$ and ${\bar{S}}_{2}$ over the vorticity against $R_{0}$. The conclusions are: (1) the in-plane residual shear $S_{2}$ was generally much larger than the non-alignment shear; and (2) the inside vortex cores ($R>20\langle R^{2}\rangle {}^{1/2}$), ${\bar{S}}_{1}/\bar{\omega }\;\approx 1.5\%$ and ${\bar{S}}_{2}/\bar{\omega }\approx 13.5\%$—i.e., the in-plane residual shear—represented 90% of the difference between the Liutex magnitude and the vorticity magnitude. It is understood that the role of the non-alignment shear is to change the direction of the Liutex vector whereas the in-plane residual shear provided energy for the rotation represented by the Liutex vector. Figure 4b infers that the direction of the vortex cores is not supposed to vary much as ${\bar{S}}_{1}/\bar{\omega }$ is small. In other words, the direction of the Liutex vector is almost the same as the direction of the vorticity vector inside the vortex cores where fluid motion behaves similar to a rigid body with in-plane residual shear.

**Figure 5:**
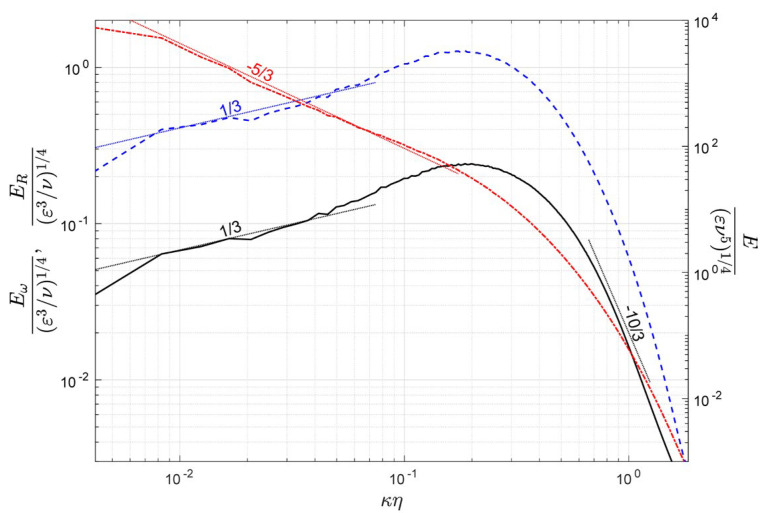
The three-dimensional energy spectrum of velocity, vorticity and Liutex vectors in homogeneous isotropic turbulence.

Investigations into turbulent structures have revealed that, instead of being purely random, many organized motions exist in turbulence, i.e., coherent structures, which are often related to vortical structures [21,22,23]. Xu et al. [14] discovered that the absolute value of the Fourier coefficients of the Liutex magnitude followed a −5/3 similarity power law, which also indicates the inherent structure of turbulence. The three-dimensional energy spectrums of the velocity, vorticity and Liutex vector are shown in Figure 5, where κ, η, ε and ν are the wavenumber, Kolmogorov scale, turbulent kinetic energy dissipation rate and kinematic viscosity, respectively. Dotted lines with powers of 1/3, −5/3 and −10/3 are added in appropriate places to compare the numerical results and the theoretical predictions. It is shown that for ideal homogeneous isotropic turbulence, the turbulent kinetic spectrum E (red dash-dotted line) follows the −5/3 law in the inertial subrange, as predicted by Kolmogorov through a dimensional analysis. The definition of the turbulent kinetic spectrum E is given as:

(11) $$k=\frac{u^{2}+v^{2}+w^{2}}{2}=\int_{0}^{\infty }E\left(\kappa \right)d\kappa ,$$

where k is the turbulent kinetic energy and u, v and w are the velocity components. According to Kolmogorov’s 1941 theory (K41), the turbulent spectrum $E\left(\kappa \right)=C\varepsilon^{2/3}\kappa^{-5/3}$. Similarly, the definition of the energy spectrum of the vorticity and Liutex could be given by:

(12) $$\frac{\omega_{x}^{2}+\omega_{y}^{2}+\omega_{z}^{2}}{2}=\int_{0}^{\infty }E_{\omega }\left(\kappa \right)d\kappa ,$$

(13) $$\frac{R_{x}^{2}+R_{y}^{2}+R_{z}^{2}}{2}=\int_{0}^{\infty }E_{R}\left(\kappa \right)d\kappa ,$$

where $\omega_{x}$, $\omega_{y}$ and $\omega_{z}$ are the vorticity components and $R_{x}$, $R_{y}$ and $R_{z}$ are the Liutex components. The energy spectrum of vorticity $E_{\omega }$ or the equivalent enstrophy spectrum—which also served as the dissipation spectrum by the multiplication of the kinematic viscosity ν—in homogeneous isotropic turbulence is also shown in Figure 5. It is verified that for incompressible HIT, $E_{\omega }$ and E satisfy the relation $E_{\omega }\left(\kappa \right)=\kappa^{2}E\left(\kappa \right)$; thus, at the same range where the turbulent kinetic spectrum E followed the −5/3 power law, $E_{\omega }$ satisfied a 1/3 power law, as shown by the blue dashed line in Figure 5. As the Liutex and vorticity had the same dimension of 1/s (per second), it was not surprising to find that the energy spectrum of Liutex also satisfied the 1/3 power law in the inertial subrange, as shown by the black line in Figure 5. The fact that the Liutex energy spectrum obeyed the 1/3 power law in the inertial subrange, however, was not trivial. We could arbitrarily introduce new quantities with the dimension 1/s; for example, ∂u/∂x and $\omega_{x}+3\omega_{x}$. Yet, we could not expect all these quantities to satisfy the given power law. Therefore, the existence of the 1/3 power law for Liutex indicated that the Liutex vector might be used to signal the flow structures in homogeneous isotropic turbulence.

**Figure 6:**
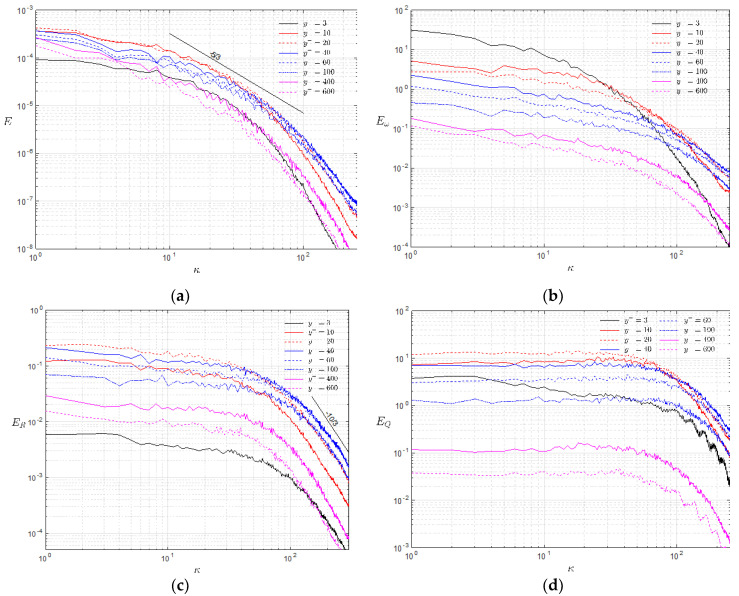
One-dimensional energy spectrum of (**a**) velocity magnitude, (**b**) vorticity magnitude, (**c**) Liutex magnitude and (**d**) Q of various distances from the wall surface in a turbulent channel flow.

A distinctive feature of the energy spectrum of Liutex is that it follows the −10/3 power law in the dissipation range, which is in accordance with previous discovery of Xu et al. [14], as they defined the spectrum as absolute values of Fourier coefficients. Even though the theoretical predictions of the −5/3 and 1/3 power laws match the numerical results of homogeneous isotropic turbulence well in the inertial subrange, it is the −10/3 power law of the Liutex energy spectrum in the dissipation subrange that persisted in wall-bounded turbulence where other quantities are generally significantly contaminated by residual shear. To illustrate this point, the one-dimensional energy spectrum of the velocity magnitude, vorticity magnitude, Liutex magnitude and Q (the parameter in the Q vortex identification method [24]) are shown in Figure 6 for a turbulent channel flow with $Re_{\tau }=950$. It is shown in Figure 6a that the turbulent kinetic spectrums only marginally match the −5/3 power law in a narrow wavenumber range. In the viscous sublayer ($y^{+}=3$), the flow there is hardly turbulent and the turbulent kinetic energy k is relatively small. Moving away from the wall surface, k quickly increases and a narrow range satisfying the −5/3 power law could be observed in the buffer layer ($y^{+}=10,20$). Further in the log-law layer, more energy is injected into the high wavenumbers in the log layer ($y^{+}=40,60,100$) where the turbulent fluctuations were the fiercest and the turbulent kinetic energy becomes smaller in the outer layer ($y^{+}=400,600$). No similarity of power law can be observed in the dissipation range. The energy of the vorticity or enstrophy, on the other hand, is relatively large even in the viscous sublayer ($y^{+}=3$), as shown in Figure 6b, due to the inclusion of both rotational motion and residual shear. The lines of the enstrophy spectrum at various heights cross each other in the dissipation range; thus, they do not obey any power-law similarity.

As shown in Figure 6c, the fluctuating energy of Liutex is negligible in the viscous sublayer because the Liutex vector is free from shear contamination. This is in the same order in the dissipation range as that of $y^{+}=600$, which is already near the center line of the channel. High energy in the dissipation range (a high wavenumber) is observed in the log layer ($y^{+}=40,60$) and, more importantly and independent of height, all the Liutex energy spectrums—except for $y^{+}=3$ in the viscous layer, where the flow could hardly be viewed as turbulence—follow the −10/3 power law in the dissipation range as in the homogeneous isotropic case. The energy spectra of Q, on the other hand, are irregular in the dissipation range, as shown in Figure 6d, and obey no power-law similarity. The zigzags of the spectral lines in Figure 6 are due to insufficient averages as only the spanwise average for one snapshot was taken. However, the general shape of the spectrum would remain the same with the average in time over many snapshots. In summary, among the 1/3, −5/3 and −10/3 power laws shown in Figure 5 for homogeneous isotropic turbulence, only the −10/3 power law of the Liutex energy spectrum in the dissipation range extends to wall-bounded turbulence.

From the discussion above, we observed that the energy spectrum of Liutex follows the −10/3 power-law spectrum in the dissipation scale range. We further hypothesized that this power law is determined by the turbulence dissipation rate ε and the kinematic viscosity ν. Through a dimensional analysis, we obtained:

(14) $$E_{R}=C_{R}\nu^{-\frac{11}{4}}\varepsilon^{\frac{19}{12}}\kappa^{-\frac{10}{3}},$$

where $C_{R}=\varphi \left(\kappa \eta \right)$ is a non-dimensional parameter. Unlike the −5/3 power-law spectrum of turbulent kinetic energy—which only exists for homogeneous isotropic turbulence and only marginally matches could be found in wall-bounded turbulence, especially with mid-to-low Reynolds numbers—the Liutex energy spectrum in the dissipation range exists even in wall-bounded turbulence of various heights; thus, this could lay a theoretical foundation for new turbulence models.

## 4. Conclusions

Compared with previous scalar vortex identification methods, the Liutex vector provides more information regarding the local rotation axis, which actually makes a difference in the quantitively analysis of the behavior of vortices in various flows, especially in turbulence. The newly discovered power-law spectrum of Liutex in a low Reynolds number turbulent boundary layer is revisited and confirmed in homogeneous isotropic turbulence with $Re_{\lambda }=315$. In this paper, we clarified that a power-law similarity exists in the viscous subrange and, in terms of the energy spectrum, the power is −10/3. In addition, the intermittency of the Liutex vector is revealed. These results indicate that the Liutex vector might be a key physical variable to describe the structures of the flow, especially turbulence, and might also lay a foundation for new turbulence modelling.

## Data Availability

Not applicable.
